# Poster Presentations - Abstracts of the 3^rd^ Brazilian Congress of PRONUCLEO and Regional Meeting Brazil Red Latinoamericana de Reproducción Asistida (REDLARA)

**DOI:** 10.5935/1518-0557.20240047

**Published:** 2024

**P-01. Frequency of diagnosed disorders in the preimplantation genetic testing for monogenic diseases (PGT-M) in Brazilian and Argentinian couples: 7 years follow up**

**L. Antunes^1^, A. Shinzato^1^, D. Lorenzi^2^, M. Di Bastiano^2^, V. Regla^1^, T.M. Ali^1^, M. Feliciano^1^, C. Dantas^1^, P.Q. Estrada^1^, B. Copreski^1^, S. Salinas^2^, M. Riboldi^1^, J.A. Martinez-Conejero^3^, A. Cervero^3^, C. Rubio^3^**

^1^Igenomix Brazil, Sao Paulo, Brazil

^2^Igenomix Argentina, Buenos Aires, Argentina

^3^Igenomix Spain, Valencia, Spain

**Objective:** To explore the nature of the conditions for which PGT- M requests have been made for Brazilian and Argentinian couples who underwent PGT-M and to compare the number of eligible embryos for transfer (low-risk and euploid) according to their inheritance pattern of the monogenic condition. Individuals or couples at increased risk of having a child with a monogenic condition may choose to mitigate this risk by seeking reproductive options such as the preimplantation genetic testing for monogenic diseases (PGT-M). Only low-risk embryos from the specific genetic condition tested are considered eligible for transfer, and if there are more than one “health” embryo they can be cryopreserved and used by the couple in future pregnancies. However, the literature offers sparse data on PGT-M for Latin America who has an admixture population, and little is known about the conditions couples requested to test for.

**Methods:** Retrospective study including 768 couples with a personal and familiar history of genetic condition, who underwent PGT-M with preimplantation genetic testing for aneuploidies (PGT-A) or structural rearrangements (SR) at Igenomix between May 2016 and January 2024. The PGT-M was carried out at Igenomix Spain. For PGT-M, a small quantity of DNA from the trophectoderm, was carried out through analysis, when possible, of the familial variant(s) and/or the previously defined SNP and/or STR markers (pre PGT-M study) using PCR fluorescent.

**Results:** In our cohort, the women age ranged from 24 to 50 years old (average 38.4 years old) and male age varied from 27 to 69 years old (average 41.4 years old). PGT-M was performed mainly on D5 trophectoderm biopsies and it was requested for at least 275 different conditions, including monogenic disorders, HLA typing and CNVs analysis (4 cases). 24 couples underwent PGT-M to assess for at least two diseases. 37 couples underwent PGT-M to select a low-risk embryo in conjunction with the HLA compatibility. An autosomal dominant (AD) disorder request was observed in 47.5% of the couples (n=365). Regarding AD conditions, in 163 cases the male was the carrier of the mutation and in 150 the female. PGT-M was carried out specially for Li Fraumeni (TP53) and Huntington disease (HTT). An autosomal recessive (AR) disorder study was made for 39.2% of the couples (n=301) mainly for sickle cell anaemia and an X-linked condition in 13.3% (n=102). Only 3 couples underwent PGT-SR due to a structural rearrangement in their karyotype. The data showed 86.85% of couples in the AD group had at least one euploid/low risk embryo for transfer; in the AR group the rate was 89.71% and for in X-linked disorders we observed 87.2%.

**Conclusion:** This is the first overview on PGT-M cases in Latin America. The data analysis has shown more requests for AR diseases than in Europe (39% vs 19%). Also, it shows the frequency of most of the disorders studied in PGT-M in the Brazilian population was very similar the previously reported by the PGD Consortium, but with higher frequency of Li-Fraumeni and sickle cell anaemia in Brazil and Argentine.

**P-02. Retrospective study of the endometrial *lactobacilli* profile of Brazilian women with recurrent implantation failure or recurrent pregnancy loss**

**V. Regla^1^, L. Antunes^1^, A. Shinzato^1^, T. Mikulski Ali^1^, M. Riboldi^1^, M. Ruiz-Alonso^2^, D.M.V. Perilla^2^, J.A. Castellón^2^**

*^1^Igenomix Brasil*

*^2^Igenomix Spain*

**Objective:** To evaluate the profile of *lactobacilli* in the endometrial microbiota of Brazilian women with recurrent implantation failure or recurrent pregnancy loss. *Lactobacillus* is the most abundant genus found in the female reproductive tract. Patients with a lactobacillus-dominant microbiota usually have one or two of the following species in higher counts: *L. iners, L. crispatus, L. jensenii and L. gasseri*, all of which except the former may play a protective role. It is known that *Lactobacilli* can help inhibit pathogenic bacterial growth and prevent dysbiosis, and a dysbiotic environment can be causative of an inflammatory uterine status leading to bad IVF outcomes such as recurrent implantation failure (RIF) and recurrent pregnancy loss (RPL).

**Methods:** This is a retrospective observational study including patients with indication for endometrial microbiome analysis between May and December 2023. Patient data were collected from EMMA/ALICE requisitions and reports, including age, body mass index (BMI), clinical indication and microbiome profile. Only first biopsies from patients with at least one *Lactobacillus* species present and absence of the pathogenic bacteria included in the test panel were analyzed. Two groups were studied regarding their endometrial lactobacillus profile: recurrent implantation failure (RIF) and recurrent pregnancy loss (RPL) patients.

**Results:** A total of 538 patients had their endometrial microbiome accessed between May and December 2023 in which 217 of them had an indication for RIF and 9 for RPL. Average age and BMI for RIF patients were 38.4 and 20.2 respectively, while for RPL patients average age was 38.4 and average BMI 30.6. Of the 217 RIF patients, 48.4% (n=105) had the presence of at least one species of *Lactobacillus* (*L. crispatus, L. gasseri, L. iners* and *L. jensenii*) and absence of the pathogenic bacteria included in the test panel, whereas of the 9 cases of RPL, 55.6% (n=5) had presence of *Lactobacillus* species and absence of pathogenic bacteria of the panel. The *Lactobacillus* analysis for RIF indicated *L. cripatus* as the most frequently detected specie, followed by *L. iners* (40.6% *L. crispatus,* 11.1% *L. gasseri,* 25.3% *L. iners* and 18.9% *L. jensenii*), very similarly, for RPL patients, both *L. crispatus* and *L. iners* were the most detected species (33.3% *L. crispatus,* 0% *L. gasseri,* 33.3% *L. iners* and 11.1% *L. jensenii*).

**Conclusion:** *L. crispatus* and *L. iners* were the most frequent *Lactobacillus* species detected in both RIF and RPL groups. This founds are in accordance with other studies where the same bacteria were in dominance. The presence of *L. crispatus* was already associated with good pregnancy outcomes and prevention of premature delivery, meanwhile the presence of *L. iners* was reported previously in disbyotic microbiotas and could be causative of disease due its clonal variants. This study is a start towards a broader understanding of the endometrial profile of *Lactobacilli* in Brazilian women and its role in pregnancy outcomes.

**P-03. Male factor infertility impacts the mosaicism rate in Day 7 blastocysts in cycles of preimplantation genetic testing for aneuploidy**

**C.D.S. Francisquini^1^, S.A. Giacomin^1^, V.B. da Rosa^2^, A. Schuffner^1^**

^1^Cínica Conceber - Centro de Medicina Reprodutiva, Curitiba, Paraná, Brazil

^2^Brown Fertility - Florida Fertility Clinics, Jacksonville, Florida, United States

**Objective**: To evaluate the euploidy and mosaicism rate of blastocysts on day 7 of development (Day 7) in outcomes of preimplantation genetic analysis for aneuploidy (PGT-A) and the association with male factor infertility.

**Methods:** Retrospective observational study including 46 Day 7 blastocysts (168h post-ICSI), from 35 PGT-A cycles, between 2013-2024. The biopsy procedure was performed on trophectoderm cells and analyzed by next generation sequencing (NGS). Inclusion criteria: fresh and homologous oocytes, fresh/cryopreserved homologous semen, *in vitro* fertilization performed by intracytoplasmic sperm injection (ICSI) and assisted hatching on Day 3. Exclusion criteria: unexplained infertility, PGT-A preceded by PGT-M and PGT-SR. The cycles were divided into two groups according to infertility factor: Group 1 (G1) - exclusively female without male factor (n=22 cycles with total of 26 Day 7 embryos) and Group 2 (G2) - male infertility associated with the female or exclusively male (n=13 cycles with total of 20 Day 7 embryos). Were compared between the groups: the rate of euploidy and mosaicism; the oocyte morphology (normal or abnormal); inner cell mass (ICM) and trophectoderm morphology (A-C; by Gardner Blastocyst Grade); and how many days the biopsy procedure was performed (1 day - Only Day 7; 2 days - Day 5 and 7 or Day 6 and 7; and 3 days - Day 5, 6 and 7). The groups homogeneity was assessed by comparing the means of female and male ages, using the ANOVA test. And the percentages were compared by Z statistical test for two proportions, adopting *p*<0.05.

**Results:** The Day 7 embryos were obtained from the IVF cycles of women aged 39.68±3.79 years (G1) and 37.61±3.22 years (G2) (*p*>0.05) and men aged 39.40±5.98 years (G1) and 42.08±7.01 years (G2) (*p*>0.05), determining the homogeneity between groups. The number of Day 7 embryos per cycle was not different (*p*>0.05) between G1 (1.2 Day 7 embryos) and G2 (1.5 Day 7 embryos), as well as the percentage was similar between Day 7 embryos that achieved initial blastocyst stage on day 5 of development (G1: 57.7% and G2: 55.0%, *p*>0.05). Mostly Day 7 blastocysts were originated from oocytes with abnormal morphology in both groups, with no statistical difference (G1: 84.6% and G2: 75.0%; *p*>0.05). There was also no difference (*p*>0.05) in relation to the ICM morphology of Day 7 blastocysts between groups G1 (A: 0%; B: 30.8% and C: 69.2%) and G2 (A: 5.0%; B: 25.0% and C: 70.0%). However, for trophectoderm classification, in the G1 (without male factor), a higher proportion (*p*<0.05) of grade C blastocysts was observed (76.9%) when compared with G2 (with male factor) (45%). It was observed in the G2 the classification B and C of trophectoderm in the same proportions (both 45.0%) and only in this group the blastocysts were graded as A for ICM or trophectoderm. In both groups, the biopsy procedure was performed predominantly within 2 days (G1: 81.8% and G2: 69.2%, *p*>0.05). In G2 (presence of male factor) most blastocysts were biopsied within 3 days (30.8%), higher than G1 (4.6%) reaching statistical significance (*p*<0.05). The euploidy rate per biopsied blastocyst was similar among the two study groups (G1: 15.4% and G2: 20.0%, *p*>0.05), however, there was a higher rate of mosaic blastocyst in G2 (with male factor) (20.0%), when compared with G1 (without male factor) (7.7%; *p*<0.05). Additionally, it was observed that Day 7 blastocysts from G2 presented a euploidy rate (20.0%) equal to the mosaic rate (20.0%), even if not statistically significant.

**Conclusion:** The results suggest that the presence of male factor infertility is associated with an increased rate of mosaicism in Day 7 embryos analyzed in PGT-A cycles.

**P-04. Obesity and Female Infertility: Possible impacts of obesity on the hypothalamic-pituitary-ovarian axis**

**M.S. Moreno^1^, B.F. Viana^2^, C.M. Ferreira^2,3^**

^1^Associação Instituto Sapientiae, São Paulo, SP, Brazil

^2^Instituto Brasileiro de Medicina e Reabilitação, Rio de Janeiro, RJ, Brazil

^3^Universidade Federal do Rio de Janeiro, Rio de Janeiro, RJ, Brazil

**Objective:** This study aims to analyze and discuss the possible consequences of obesity on female fertility, focusing on the disruption of the hypothalamic-pituitary-ovarian (HPO) axis due to hormonal dysregulation.

**Methods:** A comprehensive literature review was conducted focusing on the dysfunctions of the hypothalamic-pituitary-ovarian axis. Articles from the past decade in English and Portuguese were sourced from PubMed and Google Scholar databases. Keywords used in the search included “Infertility,” “Obesity,” “HPO axis,” “FSH function,” “LH function,” and “female.”

**Results:** The female reproductive system, governed by the hypothalamic-pituitary-ovarian axis, is intricately regulated by hormonal interactions. Obesity disrupts this axis, primarily affecting hormones such as leptin, adiponectin, and insulin due to increased adipose tissue. These disruptions lead to anovulation and alterations in follicular development, impacting fertility. Leptin, adiponectin, and insulin play crucial roles in modulating the HPO axis, affecting gonadotropin-releasing hormone (GnRH) pulsatility, folliculogenesis, and ovulation. Moreover, insulin resistance, a common feature of obesity, exacerbates hormonal imbalances, leading to further complications in female reproductive health.

**Conclusion:** The findings underscore the critical role of a well-functioning hypothalamic-pituitary-ovarian axis in female fertility. Obesity-induced disruptions in hormonal regulation, particularly involving leptin, adiponectin, and insulin, contribute significantly to infertility by impairing ovulation and follicular development. Strategies aimed at weight loss and lifestyle modifications have shown promise in improving fertility outcomes in obese women. Addressing these hormonal imbalances through targeted interventions may offer novel approaches to mitigate infertility associated with obesity.

**P-05. An increased body mass index negatively affects the top quality blastocysts rate**

**M.L. dos Santos^1^, N.P. de Oliveira^1^, C.G. Dutra^1^, N. Frantz^1^, G.M. Andrade^1^**

^1^Nilo Frantz Reproductive Medicine - Porto Alegre, Rio Grande do Sul - Brazil

**Objective:** Metabolic disorders, as inflammation and oxidative stress, due to obesity alter the oocyte’s microenvironment and impact oocyte quality and embryo development. The purpose of this study was to correlate the body mass index (BMI) and its consequences on oocyte recovery, oocyte maturity and IVF laboratory outcomes.

**Methods:** This is a single-center retrospective cohort study including 1548 IVF cycles from 2019 to 2022. Patients were divided into four body mass index (BMI) categories groups, according to the World Health Organization: underweight (≤ 18.5 kg/m^2^, n=24), health (18.5-24.9 kg/m^2^, n=985), overweight (25.0 and 29.9 kg/m^2^, n=389) and obese (≥ 30.0 kg/m^2^, n=150). Patients and cycle characteristics as age, AMH dose, FSH and LH dose and days of medication were compared among the groups. Laboratory outcomes as oocyte recovery, oocyte status of maturation, blastocyst, top quality blastocysts and euploidy rates were compared between groups. Statistical analyses were performed using GraphPad. A one-way ANOVA test was used to compare BMI groups with a post hoc test (Fisher’s) when necessary. The chi-squared test was used to compare proportions, *p*-value ≤ 0.05 was considered statistically significant.

**Results:** The average age of the groups ranged from 35.4 and 36.7 years. Interestingly, the oocyte recovery rate was lower in the healthy (73.2%) and obese (72.7%) groups when compared to the overweight (75.3%). No differences were observed regarding the oocyte metaphase II rate but the overweight group (9.5%) had an increased rate (*p*=0.0002) of oocyte on germinal vesicle (GV) stage compare to health group (11.5%). The overweight (*p*=0.04) and obese (*p*=0.04) patient groups have an decreased top quality blastocyst rate compare to underweight group, being this difference observed also when we compare the overweight group with the healthy group (*p*=0.06). There was no difference between the groups regarding the others laboratory outcomes evaluated as fertilization, total blastocyst and euploidy rates.

**Conclusion:** These findings support the concept that metabolic disorders caused by obesity impair meiotic and cytoplasmic maturation of the oocyte which reduces its developmental competence for fertilization and blastocyst development and also the quality of these embryos.

**P-06. Cannabidiol (CBD) and ∆9-Tetrahydrocannabinol (THC): A review study on the therapeutic potential of substances in the signs and symptoms of endometriosis**

**J.R. Godoy^1^, J.P. de Castilhos^1^, F.B. Caruso^2^, N.F. de Vasconcelos^1^, M.R. Hentschke^1, 3^**

^1^PUCRS - Porto Alegre - RS - Brasil

^2^UFRGS - Porto Alegre - RS - Brasil

^3^Fertilitat - Medicina Reprodutiva - Porto Alegre - RS - Brasil

**Objective:** To study and discuss the therapeutic potential of the two main cannabinoids from the *Cannabis sativa* plant, Cannabidiol (CBD) and ∆9-tetrahydrocannabinol (THC), in the signs and symptoms of patients with endometriosis.

**Methods:** Bibliographic review with active search on the PubMed, Scopus and Embase platforms, with descriptors established through DeCS/MeSH for the first base and controlled vocabulary consultation for the other two. After analyzing all the articles found, 9 articles related to the research topic were obtained.

**Results:** Studies carried out *in vitro* and *in vivo* demonstrated the relationship between the endocannabinoid system and endometriosis, reporting important therapeutic properties activated by CBD and THC. A large study conducted in an animal model, through surgical induction of endometriosis, aimed to investigate the effect of CBD on the disease and revealed anti-inflammatory, antiangiogenic and antioxidant properties of the substance, as well as demonstrating a reduction in the area of implanted lesions. Finally, self-reported studies in humans were analyzed, involving various topics related to the use of Medicinal Cannabis by patients with the disease.

**Conclusion:** The work carried out to date points to the therapeutic potential of the cannabinoids THC and CBD in the treatment of chronic pelvic pain, inflammation, cell proliferation and other symptoms associated with endometriosis. However, carrying out new clinical trials is essential to validate their efficacy and safety in human patients, including the evaluation of possible side effects, dosages, routes of administration and other aspects. Currently, some clinical trials are underway, such as NCT05670353 (São Paulo, Brazil) and NCT04527003 (Pennsylvania, United States of America), which reaffirms the need to maintain and improve research on this topic.

**P-07. Correlation between oocyte pickup-denudation time interval and blastocyst formation**

**C. Kroll^1^, K.N. Cypriano^1^, S. Sfeir Filho^1^**

^1^Fertilizar Centro Catarinense de Reprodução Humana

**Objective:** Denudation is a necessary technique for performing *in vitro* fertilization through the use of intracytoplasmic sperm injection (ICSI). The objective of this work was to verify whether there is a correlation between oocyte pickup (OPU)-denudation time interval and number of blastocyst formed.

**Methods:** This is a cross-sectional study. Patients who underwent OPU to perform the ICSI technique in 2023 in a clinic in the southern region of Brazil were included in the study. All ICSI cycles included the OPU of at least one oocyte in metaphase II and own or donor semen. The outcomes analyzed in this study were the time (minutes) of incubation of oocytes from OPU to denudation technique and the quantity (number) of blastocysts formed and quantity (number) of blastocysts considered to be of high-quality (AA, AB ou BA, Gardner grade). Cases in which the information was not completely available were excluded.

**Results:** A total of 34 ICSI cycles were analyzed. The minimum incubation time was 20 minutes and the maximum was 178 minutes, with a median of 60 minutes (interquartile range 24 minutes). Spearman’s correlation analysis (Table 1) demonstrated a negative correlation between the OPU-denudation time interval and the number of blastocysts formed (-0.384, *p*=0.025, n=34) and the number of high-quality blastocysts formed (-0.390, *p*=0.030, n=31).

**Conclusion:** There is a negative correlation between between the OPU-denudation time interval and the number of blastocysts formed. The shorter the incubation time until denudation, the greater the quantity of blastocysts and high-quality blastocysts formed. More studies are needed.

**Table 1:** Spearman’s correlation analysis between OPU-denudation time interval and number of blastocyst and high-quality blastocyst formed

	Spearman rho and *p* value		
	OPU-denudation time interval	N. of Blastocyst	N. of high quality blastocyst
OPU-denudation time interval	1	-0.384; *p*=0.025	-0.390; *p*=0,030
N. of blastocyst		1	0.619; *p*<0.000
N. of high quality blastocyst			1

OPU: oocyte pickup

**P-08. Correlation Between Sperm DNA Fragmentation and Spermogram Parameters**

**B.T. de Paula^1^, R.L.L.S. Alvarenga^1,2^, M.S. Procópio^3^**

^1^Cegonha Medicina Reprodutiva

^2^Fertitech Tecnologia em Reprodução Assistida

^3^Clinica Ovular

**Objective:** The sperm DNA fragmentation test is an additional test to evaluate the male factor. However, there is still no consensus on its real applicability. The aim of this study was to analyze the correlation between sperm DNA fragmentation at three cutoff points and spermogram parameters: age, volume, pH, total motility, progressive motility, bacterial flora and sperm morphology.

**Methods:** A spermogram and DNA fragmentation test were performed on 149 samples from patients undergoing infertility treatment at Clínica Cegonha Medicina Reprodutiva. For statistical analysis, the data were subjected to the D’Agostino & Pearson normality test and after determining the sample distribution, correlation analyzes were performed using the Spearman test.

**Results:** The results show that there is a correlation between sperm DNA fragmentation and spermogram parameters (pH, motility, progressive motility and morphology). Furthermore, another important information could be extracted from our results. We observed that 8% of the samples that presented all parameters within normal limits according to the WHO presented abnormal levels of sperm DNA fragmentation.

**Conclusion:** At the cutoff points studied, the correlation between fragmentation of 15% and above 30% indicates that changes such as pH and motility are interdependent with the increase in fragmentation of sperm DNA. Finally, it was possible to state that 8% of our entire patient population would be incorrectly diagnosed as normal spermograms, but had altered DNA fragmentation levels.

**P-09. Poor ovarian response: What are the implications for developmental and genetic health of embryos?**

**L.T.S. Rodrigues^1^, M.M. Piccolomini^2^, O. Duarte^2^, E.G. Lo Turco^1,3^, L. Yamakami^2^, A.L. Faria^2^, F.P. Ferreira^1,3^**

^1^Urology Department, Human Reproduction Section, São Paulo Federal University, São Paulo, Brazil

^2^Lab for Life, São Paulo, Brazil

^3^Neo Vita Clinic, São Paulo, Brazil

**Objective:** Low ovarian response is characterized by a reduction in the follicular response resulting in a reduced number of eggs. This fact has an impact on the outcome of assisted reproduction treatments. Therefore, understanding the genetic behavior of the embryos of these patients is still a great challenge for both the clinic and the *in vitro* fertilization laboratory, because, with a better understanding of the embryo’s genetic segregation process, it will be possible to apply more assertive behaviors in the human reproduction routine. This study aimed to elucidate the impact of the low ovarian response on embryonic development and the genetic normality rate of embryos.

**Methods:** This retrospective cross-sectional study included 73 patients up to 35 years old, splitted into two groups. The poor ovarian responder group (n=32) and the controls (n=41). The study group included patients with poor ovarian response, with less than six MII oocytes after ovarian pickup. The control group was selected according to the following inclusion criteria: the presence of tubal factor unexplained infertility, or adenomyosis. The data were collected between January 2019 and December 2021. All patients included in the study (n=73) underwent the same assisted reproduction treatment with embryos transferred after biopsy and PGT-A. The T-Student test was applied to compare numerical variables and the q-square test was for categorical variables. All the analysis was performed in SPSS software (V26). Patients who had other factors associated with poor ovarian response were excluded.

**Results:** In the comparison between the groups, the differences in the levels of AMH, progesterone, and the number of oocytes in MII were observed, as expected for the two groups (*p*<0.05). However, the control group had a higher rate of chromosomally normal embryos when compared to patients with a low ovarian response (60.9%±30.1 *vs*. 37.6%±39.7, *p*=0.0057) respectively. The other variables such as age, FSH, LH, BMI, estradiol, fertilization rate, rate of blastocyst formation, and quality of blastocysts did not show statistically significant differences.

**Conclusion:** Patients with a low ovarian response may have lower rates of embryonic chromosomal normality, this fact is important for the indication of genetic testing for these couples. Future prospective studies should be carried out with a larger number of patients to understand the mechanisms that cause genetic alterations in embryos.

**P-10. Observing the clinical results by kid-score comparison in embryos from imported oocytes**

**V.D Trindade^1^, R. Azambuja^1^, F. Wingert^1^, M.R. Hentschke^1^, V.C. Dornelles^1^, N. Vasconcelos^1^, L. Okada^1^, A.Petracco^1^, M. Badalotti^1^**

^1^Fertilitat - Reproductive Medicine Center, Porto Alegre, Brazil

**Objective:** To evaluate if the KIDScore is associated with clinical and laboratory outcomes using oocyte donor cycles from imported eggs.

**Methods:** Retrospective, observational study performed at a reproductive medicine center, using data collected between 2021 and 2023. All patients performed ICSI using imported eggs. A total of 217 blastocysts cultivated in a time-lapse incubator (Embryoscope^®^, Vitrolife^®^) were included. Embryos were stratified by KIDScore: G1: 0.1-3.9 (n=51); G2: 4.0-6.9 (n=89) and G3: 7.0-9.9 (n=77). The t-test, ANOVA, chi-square test were used for analysis, considering *p*<0.05.

**Results:** When comparing G1 *vs*. G2 *vs*. G3, the following results were found, respectively: male age (45.4 *vs*. 42.4 *vs*. 42.7, *p*=0.243), female age (43.5 *vs*. 42.8 *vs*. 43.1, *p*=0.525), egg donor age (24.4 *vs*. 24.7 *vs*. 23.7, *p*=0.247), fertilization rate % (77.3 *vs*. 77.4 *vs*. 81.1, *p*=0.710), blastulation rate % (29.2 *vs*. 47.3 *vs*. 60.8, *p*< 0.0001), implantation rate % (31.1 *vs*. 52.7 *vs*. 51.3, *p*=0.017), clinical pregnancy rate % (37.3 *vs*. 53.9 *vs*. 48.0, *p*=0.162). When comparing G1 *vs*. G3, the following results were found, respectively: male age (*p*=0.027), implantation rates (*p*=0.016), clinical pregnancy rate in frozen embryo transfers (*p*=0.034). When comparing G1 *vs*. G2, implantation rates were significantly different (*p*=0.008).

**Conclusion:** Lower kidscore was associated with higher male age and with lower implantation rates. A tendency to higher pregnancy and clinical pregnancy rates were observed in the group with higher kidscore. More studies including larger samples should be performed for better conclusions.

**P-11. Patient body mass index does not influence the incidence of vacuoles in oocytes**

**C.G. Dutra^1^, N.P. de Oliveira^1^, G. Frantz^1^, Nilo Frantz^1^, G.Mamede Andrade^1^**

^1^ Nilo Frantz Reproductive Medicine - Porto Alegre, Rio Grande do Sul - Brazil

**Objective:** Oocyte vacuolization is a common observation in clinical IVF. Metabolic disorders due to obesity alter the oocyte’s microenvironment and impact oocyte quality. The purpose of this study was to correlate the body mass index (BMI) and the incidence of oocyte vacuoles and its consequences on laboratory outcomes.

**Methods:** This is a single-center retrospective cohort study including 80 IVF cycles from 2019 to 2022. Patients were divided into four body mass index (BMI) categories groups, according to the World Health Organization: underweight (≤18.5 kg/m^2^), health (18.5-24.9 kg/m^2^), overweight (25.0 and 29.9 kg/m^2^) and obese (≥30.0 kg/m^2^). Patients and cycle characteristics as age, AMH dose, FSH and LH dose and days of medication were compared among the groups. The incidence of oocyte vacuoles were blind evaluated by two senior’s embryologists, through oocyte denuded photos before the ICSI procedure. Additionally, laboratory outcomes as recovery, maturation, blastocyst, top quality blastocysts and euploidy rates were compared between groups. Statistical analyses were performed using GraphPad. A one-way ANOVA test was used to compare BMI groups with a post hoc test (Fisher’s) when necessary. The chi-squared test was used to compare proportions, *p*-value ≤ 0.05 was considered statistically significant.

**Results:** Were evaluated a total of 704 oocytes. The presence of macro vacuoles were identified in low proportion of oocytes (2.56%), ranging from 1.18% of incidence in the healthy group until 3.91% in the obese group. Although the numerically difference no statistical association was found between the presence of vacuoles in oocytes and the patients’ body mass index. Additionally, no difference between the groups regarding the laboratory outcomes as fertilization, blastocyst and euploidy rates was observed.

**Conclusion:** Although obesity is associated with metabolic changes in oocytes and previously observed by our group associated with the oocyte SERC incidence, the present study shows that the patient body mass index is not statistically associated with the presence of vacuoles in oocytes. Larger and automated studies are important to validate the results.

**P-12. Preservation of female fertility: The search for oocyte vitrification**

**L.K. Nishikawa^1^, J.L. Nunes^1^, N.I. Ceschin^1^, A.P. Ceschin^1^, P.C. Kreling^1^, O.A.A. Goitia^1^**

^1^Feliccità Instituto de Fertilidade

**Objective:** To demonstrate the social oocyte cryopreservation data from our Institute, and analyze their growth.

**Methods:** Patients who underwent follicular aspiration and cryopreserved their oocytes with the aim of postponing pregnancy, from 2017 to 2023, were included in this study. The number of cycles performed, the number of cryopreserved mature oocytes and the age of the patients were evaluated. The patients were divided into two groups, with the COVID-19 pandemic as the dividing line. Group 1: patients who underwent the procedure from 2017 to 2020, and Group 2: patients who underwent the procedure from 2021 to 2023.

**Results:** During the evaluated period, a total of 172 cryopreservation cycles were carried out, obtaining 1.088 mature oocytes. In group 1, 58 cycles were performed, obtaining 338 mature oocytes for cryopreservation, with a mean age of 36.1 years. In group 2, 114 cycles were performed, with vitrification of 750 mature oocytes and a mean age of 35.5 years. Based on the data obtained, there were a 98% increase in demand for the proceedment. The average age of patients remained close, decreasing by 0.6 years. The number of vitrified oocytes increased proportionally.

**Conclusion:** The data collected in this study indicate that an increasing number of women are seeking oocyte cryopreservation, with the aim of postponing pregnancy. The social oocyte preservation is a good ally for those patients seeking the method before fertility declines, increasing the chances of a future pregnancy with their genetic material. The increasing publicity of the technique in the media, the uncertain post-pandemic period and the search for female reproductive autonomy are some factors that contribute to the increase of the demand.

**P-13. Add-Ons: Can chat-GPT provide evidence-based information to patients?**

**M.M. Carneiro^1^, R. Bossi^1^, A.C. Goulart^1^, M.M. Carneiro^2^, M. Sampaio^1^**

^1^Origen Centro de Medicina Reprodutiva, Belo Horizonte-MG, Brazil

^2^Faculdade de Letras da UFMG

**Objective:** Add-ons are extra procedures used to improve pregnancy rates in IVF at an extra-cost. The number of people that search online for health information keeps increasing. Chatbots such as chat GPT can answer questions and may be used by people needing help with their fertility treatments. Thus we aimed to ask ChatGPT questions about the use of add-ons and determine the quality and reliability of the answers provided.

**Methods:** GPT version 3.5 answered questions based on the Human Fertilisation & Fertility Authority add-on information for: assisted hatching (AH), preimplantation genetic testing for aneuploidies (PGT-A), embryo glue, endometrial scratching, intralipid, time lapse, Plasm rich-platelet (PRP), IMSI, Zymot, PICSI, intravenous immunoglobulin(IVG) and the ERA test. For each add-on we asked chat GPT to tell us about the add-on . Two authors used the DISCERN tool to check the quality and reliability of answers. DISCERN comprises 16 questions divided into 3 sections: reliability (1-8); quality of information on treatment choices ( 9-15) and overall rating (16). Each question was scored from 1 to 5 ( 1 = no agreement ; 5 = highest agreement ; minimum score: 16; maximum: 80). The information was evaluated by two of the authros (RB and MMC). Divergences were setteld among authors.This study is exempt from ethical approval because it did not include any interaction or intervention with human subjects .

**Results:** Chat GPT version 3.5 was good for questions related to the aims of the procedure and shared-decision making whereas it did not provide additional sources of information nor was it clear on the sources it used to answer questions. Nweither did it provide additional sources of support. It admitted there were areas of uncertainty regardin the use of add-ons. The DISCERN score ranged from 41 to 48 (mean: 43,5) out of 80 (54,3%) which is considered a “fair” score. Zymot achieve the highest score (48) followed by ERA, AH and IVG with 45. No further comparisons were possible with the answers chat GPT 3.5 provided.

**Conclusion:** Chat GPT 3.5 performs fairly when informing about the reliability and the quality of the 12 add-nos included in this study. As add-ons represent quite a controversial area for which patients often look for accurate and reliable information, they should be aware although chat GPT is a tool in constant development, it is not reliable to help guide their decisions on add-on treatments.

**P-14. Comparative analysis of fresh and frozen-thawed oocytes *in Vitro* Fertilization outcomes**

**D. Bastos^1^, R. Aguiar^1^, R. Koike^2^, E. Morita^2^**

^1^Divisão de Embriologia CITI Hinode

^2^Divisão Médica CITI Hinode

**Objective:** The study aimed to evaluate the embryonic development and pregnancy rate of frozen-thawed oocytes compared to fresh oocytes in patients undergoing *in vitro* fertilization (IVF).

**Methods:** A retrospective study was conducted on cases of frozen-thawed and fresh oocytes treated at the CITI Hinode clinic in São Paulo, from January 2022 to December 2023. Inclusion criteria considered patients undergoing intracytoplasmic sperm injection (ICSI) with own or donated oocytes. The selection of the frozen-thawed oocyte group included only cases where oocytes were frozen at the clinic. A total of 508 patients were included in the study, with 427 undergoing fertilizations with fresh oocytes and 81 with frozen-thawed oocytes. Data from 787 thawed oocytes were collected, of which 755 oocytes survived, with a survival rate of 95.9%. Fertilization rates, cleavage rates, top-quality cleavage rates, blastocyst formation rates, top-quality blastocyst rates, and positive beta-hCG rates were evaluated. Cleavage rates were assessed on the third day of development, classified according to the number of cells and fragmentation, and blastocyst rates on the fifth, sixth, and seventh days of development according to the Gardner criteria. Evaluation of beta-hCG was performed nine days after transfer, including only cases of thawed embryos transferred subsequently.

**Results:** The fertilization rate showed a significant difference between the groups, with means of 85.3% for fresh oocytes and 78.9% for frozen-thawed oocytes (*p*<0.01). Regarding the cleavage rate on the third day of development, fresh oocytes had a mean of 88.7%, while frozen-thawed oocytes had 86.6% (*p*=0.035). Fresh oocytes also showed better performance in the top-quality cleavage embryo category, with a mean of 66.5%, compared to 61.2% of frozen-thawed oocytes (*p*=0.038). Regarding blastocyst formation rate, there was no significant difference between the groups, with means of 61.4% for fresh oocytes and 61.6% for frozen-thawed oocytes (*p*=0.538). However, the top-quality blastocyst rate was significantly higher in the frozen-thawed oocyte group, with a mean of 66.7%, compared to 52.3% in the fresh oocyte group (*p*<0.001). Regarding the positive beta-hCG rate after transfer of thawed embryos, there was no difference between the groups (75.9% for fresh oocytes and 75.0% for frozen-thawed oocytes, *p*=0.888). This result demonstrates that embryos thawed from thawed oocytes may have the same chance of successful implantation. Although fresh oocytes outperform in certain aspects, such as fertilization, cleaved embryos, and high-quality cleavage-stage embryos, both fresh and frozen oocytes have shown a significantly higher proportion of high-quality blastocysts. Despite this difference, all values found between the two groups are above the benchmarks.

**Conclusion:** Therefore, our study shows that the use of frozen or fresh oocytes has no significant negative impact on treatment. The results of this study indicate that the use of fresh or frozen oocytes can be considered safe during *in vitro* fertilization treatment.

**P-15. Successful Live Birth Outcome from Euploid Embryo Derived from 3PN Fertilized Oocyte: A Case Report**

**R. Aguiar^1^, D. Bastos^1^, R. Koike^2^, E. Morita^2^**

^1^Divisão de Embriologia CITI Hinode

^2^Divisão Médica CITI Hinode

**Objective:** This case report highlights the successful pregnancy and live birth achieved through the transfer of a chromosomally normal embryo originating from an oocyte fertilized with three pronuclei (3PN), following comprehensive ploidy analysis.

**Methods:** A 40-year-old patient underwent *in vitro* fertilization (IVF) with donated oocytes and donor sperm. Fertilization assessment occurred post-ICSI, with subsequent embryo development monitored and evaluated. Biopsy and pre-implantation genetic testing for aneuploidies (PGT-A) were conducted to select euploid embryos for transfer.

**Results:** Among 12 fertilized oocytes, one displayed 3PN and developed into a high-quality euploid embryo. On the third development day, the 3PN-derived embryo displayed 14 cells with <10% fragmentation. On the fifth day of development, four embryos reached blastocyst level for biopsy, including one originating from a 3PN fertilized oocyte. This embryo received a 5AB grading according to Gardner’s criteria, indicating the best morphological classification compared to other blastocysts. Notably, one of the euploid embryos was derived from a 3PN oocyte, and genetic analysis revealed it to be a diploid embryo with a mitoscore of 19.01 The decision to transfer this specific euploid embryo was based on its superior quality compared to the others. This embryo was transferred to a surrogate uterus, resulting in successful implantation and pregnancy. Serial monitoring via beta-HCG measurements and ultrasound examinations confirmed normal fetal development. The baby was born after a full-term delivery a little over two months ago, weighing 2.950 kg and measuring 48 cm, with Apgar scores of 8/9. Presently, the baby’s weight has increased to 5.085 kg, and the length has extended to 56 cm, indicating healthy growth and development.

**Conclusion:** This case underscores the potential of 3PN-derived euploid embryos in achieving successful pregnancies, warranting further investigation into their impact on IVF outcomes.

**P-16. Retrospective analysis of assisted hatching procedure application in thawed embryos**

**J. Boncristiano, E.C.Garavini^1^, B.R.L. Moura^1^, L.C.C Evangelista^1^**

*^1^Gerar InVitro*

**Objective:** Comparison of results between embryos subjected to the Assisted Hatching (AH) procedure - Study Group - and embryos not subjected - Control Group - after thawing, in the rates of ßhCG and Clinical Pregnancy.

**Methods:** During the study period, 1255 embryo transfers were conducted, all involving thawed embryos, between January 2021 and November 2023. Participants were stratified into four distinct age groups (<35 years, 35 to 39 years, 40 to 42 years, and >42 years), reflecting a broad age distribution. The average age of patients was 36.7 years. Oocytes were fertilized using the Intracytoplasmic Sperm Injection (ICSI) technique, and Assisted Hatching was performed using a Partial Zona Pellucida Dissection (PZD) needle. Transfer results were recorded in a spreadsheet, where the Chi-Square test was employed to analyze statistical significance between age groups, allowing for a comprehensive assessment of differences and similarities in reproductive success rates in each specific age bracket.

**Results:** In the results, in the control group with 662 transfers without Assisted Hatching (AH), the positive ßhCG rate was 44.1%, while in the study group with 593 transfers undergoing the AH procedure, the rate was 41.3%. No statistically significant differences were observed between the groups (*p*=0.31). Regarding clinical pregnancy, in the control group (640 transfers), the rate was 28.4%, and in the study group (553 transfers), the rate was equally 28.4%. Once again, no statistically significant differences were found between the groups (*p*=0.99). Additionally, participants were divided into four age groups (<35 years, 35 to 39 years, 40 to 42 years, and >42 years). Patients under 35 undergoing Assisted Hatching (AH) showed a clinical pregnancy rate of 27.4%, while those not undergoing the procedure recorded a rate of 34.5%. For patients aged 35 to 39, the group undergoing AH achieved a clinical pregnancy rate (CPR) of 34.4%, compared to 23.9% in the non-submitted group. Patients aged 40 to 42 in the AH group had a positive CPR of 20.2%, while in the non-submitted group, the rate was 28.2%. Patients aged over 42 undergoing AH had a positive CPR of 27.3%, while in the non-submitted group, it was 20.3%. These results highlight the influence of age on clinical pregnancy rates, revealing significant variations in age groups undergoing or not undergoing the AH procedure.

**Conclusion:** The results indicate that Assisted Hatching (AH) did not significantly impact overall rates of positive ßhCG and clinical pregnancy. However, age-specific analyses revealed nuances. Patients under 35 undergoing AH showed a slightly lower clinical pregnancy rate, while those between 35 and 39 benefited. For women aged 40 to 42 and above 42, AH demonstrated lower rates. The influence of AH varies with age, suggesting its application should be considered carefully across different age groups.

**P-17. Sperm separation with sex selection through density gradient centrifugation in cycles with PGT-A**

**P.I.N. Huamán^1^, Y.E.O. Acevedo^1^, L.S.B.D.^1^, C.G.H. Córdova^1^, C.Z. Moreno^1^, J.S. Agurto^1^, J.Q. Prado^1^, J.P. Ruiz^1^**

^1^Instituto de Medicina Reproductiva Clínica Ricardo Palma

**Objective:** To evaluate the efficacy of the sperm separation method with sex selection through density gradient centrifugation in couples undergoing IVF/ICSI procedures with PGT-A.

**Methods:** A retrospective study was conducted between January 2018 and December 2022 involving 29 cycles of IVF/ICSI with PGT-A that requested sex selection in the preparation of the semen sample at the Ricardo Palma Clinical Reproductive Medicine Institute, Lima, Peru. Patients signed an informed consent agreeing that the sperm separation technique with density gradients does not guarantee the selection of the desired sex. Sex selection was performed using density gradients (Fertipro, Belgium). At the bottom of a conical tube, 2 ml of lower layer was placed, followed by 2 ml of upper layer, and 1 - 2 ml of semen at the top. It was centrifuged for 20 minutes at 200g. Ideally Y-bearing sperm were recovered from the upper layer, and ideally X-bearing sperm from the pellet of the lower layer. Fisher’s Exact tests were used to compare the proportion of female/male ratio in each sex selection. *p* values <0.05 were considered statistically significant.

**Results:** 16 couples requested male sex selection, and 76 embryos were analyzed, with 50% being male. While in the 13 couples that requested female sex selection, the proportion of female embryos was 52% out of 50 embryos analyzed. No significant differences were found in the proportions of sexed embryos in each group. Furthermore, an analysis was conducted by IVF or ICSI technique in the proportion of embryos with sex selection. No significant differences were found by insemination technique.

**Conclusion:** The sperm separation technique through density gradient centrifugation for sex selection does not guarantee obtaining an embryo of the requested sex.

**P-18. Impact of “Gender Affirmation Therapy” on the fertility of transgender people**

**P.H.M. Marques^1^, M.G.L. Rocha^1^, M.L.P.C. da Silva^2,3^, M.R. Tolentino^3^, R.M. Lamaita^1,3^**

^1^Faculdade de Farmácia UFMG

^2^Pontifícia Universidade Católica (PUC)

^3^Rede Mater Dei de Saúde

**Objective:** Transgender people (TP) are those whose gender identity differs from the gender attributed to them at birth due to biological sex. Among that community, it is common the desire for body modification that helps their gender expression to match their gender identity, by means of surgery or aesthetic procedures, or through “gender affirming therapy” (GAT). However, these “therapies” might present a major impact in reproductive life. The primary objective of the study is to impart knowledge regarding the impact of “gender-affirming therapies” on the reproductive capacity of transgender patients. The main focus is on raising awareness among professionals and patients to ensure these individuals have the opportunity to build their families

**Methods:** Hence, a research in literature was made, selecting papers that brought the relevance of “GAT” in PT lives. In addition, the side effects are also described, especially the impacts of this therapy on the testicles and ovaries, in the histology and structure of these organs, as well as the consequences on gametogenesis and on the quality of oocyte and spermatozoa.

**Results:** To cease the “GAT” may lead to a reestablishment of the reproductive capacity, for people with ovaries, a restart of menses and ovulation, while for people with testicles there might be an improvement of semen parameters. Nevertheless, none of these studies made a long time analysis of PT on use of “TAG”, for example 10 years, which represents a limitation. Finally, fertility preservation (PF) options are presented in different stages, such as gametes or tissue freezing, according to the protocol adopted.

**Conclusion:** Therefore, Assisted Reproduction has solid techniques to meet a portion of PT that seek for PF, via semen freezing or oocyte vitrification, but other demands, such as freezing of testicular and ovarian tissue, are still below the ideal. Moreover, this health field is still very elitist, and flees the reality of the general population, and equally, the reality of PT, particularly in Brazil.

**P-19. The impact of endometriosis on embryo development and ploidy**

**S.M. Jau^1^, M.L. Montenegro^1^, L.T.S. Rodrigues^2^, C.S. Souza^1^, R. Tomioka^3^, A.M. Alvarenga^3^, E.G. Lo Turco^2^, F.P. Ferreira^1^**

*^1^Neo Vita Clinic, São Paulo, Brazil*

*^2^Urology Department, Human Reproduction Section, São Paulo Federal University, São Paulo, Brazil*

*^3^Lab for Life, São Paulo, Brazil*

**Objective:** The study aims to assess the impact of endometriosis on the proportion of genetically normal embryos.

**Methods:** In this retrospective cohort study, we included 328 patients, divided into a study group (n=181) and controls (n=147). Data were collected between January 2019 and December 2021. The study group included patients with endometriosis without any other associated infertility factors. Controls were selected according to the following inclusion criteria: the presence of tubal factor, unexplained infertility, or presence of adenomyosis. All patients included in the study received the same controlled ovarian stimulation protocol and underwent the same assisted reproduction treatment with embryos transferred after the Preimplantation Genetic Test for aneuploidy (PGT-A). Statistical analysis of the data was performed using Student’s T-test and Chi-square test with SPSS software (V26). And an alpha of 5% was considered for the difference between the groups.

**Results:** In the comparison between groups, the variables age, LH, FSH, AMH, Estradiol, Progesterone, fertilization rate, rate of development to blastocyst, rate of chromosomally normal embryos, and rate of embryo implantation, did not show statistical difference. However, patients with endometriosis had a lower rate of stage MII oocytes (0.73±0.32 *vs*. 0.87±0.45, *p*<0.002).

**Conclusion:** Endometriosis is a chronic disease and one of the main causes of female infertility that affects thousands of women worldwide. Many articles relate to endometriosis implantation failures and poor oocyte quality. However, the relationship of endometriosis with embryonic development and possible genetic changes in these embryos is not well established. Thus, in this study, no relationship was observed between endometriosis and embryonic development and endometriosis and chromosomal changes in embryos. Moreover, we observed a relation between endometriosis and the oocyte rate in metaphase II when compared with the control group.

**P-20. Sample stability for DNA Fragmentation Index evaluation**

**P.R.P. dos Reis^1^, M. Silva^1^, G.C.S. Chaves^1^, R.C.R. Andreolli^1^, A.O. dos Santos^1^, R.M.C. Penteado^1^, A.A.R. Villarinho^1^, J.C.C. Guerra^1^**

^1^Laboratório de Patologia Clínica do Hospital Israelita Albert Einstein

**Objective:** Infertility affects around 280 thousand couples in Brazil, and even though without a defined cause, the genomic quality of spermatozoa plays a prominent role. Even with normal mobility and morphology, spermatozoa may have DNA damage. DNA fragmentation testing in this context is important in aiding infertility investigations, such as in sample selection for *in vitro* fertilization, as it assesses the percentage of fragmented/damaged spermatozoa. Our laboratory routine utilizes fresh samples; however, the possibility of using frozen semen is a significant milestone for laboratory routines, as it allows workflow optimization and facilitates receiving samples from services distant from the laboratory. In this scenario, the objective of the study was to verify the stability of samples after freezing for performing the DNA Fragmentation Index test.

**Methods:** Twenty-one routine semen samples underwent DNA fragmentation testing at four time points: fresh, 1 day, 4 days, and 8 days after freezing and thawing. Within one hour after the collection, the fresh sample was submitted to the DNA fragmentation test, after that, it were subjected to freezing at -80^o^C. The Sperm Chromatin Dispersion (SCD) Method was used to evaluate the DNA fragmentation index. The kit was developed “in-house”, respecting all the necessary steps: pre-treated slide, sample insertion into agarose tube, thermal treatment steps, homogenization, application of denaturing and lysing solutions, specific quantification and staining. Then, 500 cells were counted, and the percentage of fragmented spermatozoa was calculated. The reference value is considered normal up to 30% fragmentation. Statistical analysis was performed using Pearson correlation test and Student’s t-test.

**Results:** There was no statistically significant difference among the samples (*p*>0.05), including was found correlation values above 0.9, regardless of the storage time in frozen form, thus ensuring sample stability for DNA fragmentation index evaluation.

**Conclusion:** The possibility of receiving frozen samples from different institutions has made this test more widely available, as its increasing applicability is evident, not only in the context of infertility, but also in the selection of semen for fertilization.

**P-21. Validation of the weighing for seminal volume measurement directly in collection container**

**P.R.P. dos Reis^1^, M. Silva^1^, G.C.S. Chaves^1^, R.C.R. Andreolli^1^, A.O. dos Santos^1^, R.M.C. Penteado^1^, A.A.R. Villarinho^1^, J.C.C. Guerra^1^**

^1^Laboratório de Patologia Clínica do Hospital Israelita Albert Einstein

**Objective:** To validate the process of weighing seminal ejaculate using an analytical balance, taking into consideration the semen density of 1g/ml. In semen analysis, evaluating ejaculate volume is one of the essential criteria for determining male reproductive health. Seminal volume, representing the quantity of ejaculated fluid, is a fundamental measure to assess the function of male sex glands. The lower reference limit for semen volume is 1.5 ml (5th percentile, 95% confidence interval (CI). Smaller or larger volumes may indicate potential fertility issues. According to the World Health Organization (WHO) 6th ed., it is recommended that seminal volume measurement preferably be done by weighing, using an analytical balance. Direct weighing of the sample in the collection container provides a more precise measure of seminal volume, as it avoids potential measurement errors associated with other methods, such as using syringes or pipettes, allowing for a standardized approach and essential for ensuring consistent and comparable results among different laboratories. Weighing the container with the ejaculate and subtracting the weight of the empty container to obtain the sample weight is a relatively simple and straightforward procedure, easily performed in laboratory settings.

**Methods:** During the period from 02/10/2023 to 23/10/2023, 123 collected semen samples were measured in graduated conical tubes and then the same materials were weighed using an analytical balance. Initially, the universal container was weighed, and the value recorded. Then, the sample was aspirated with a Pasteur pipette and transferred to a graduated conical tube to determine the volume. After this step, the empty container was weighed again, and the value recorded. The results of both measurements, conical tubes and analytical balance, were evaluated through statistical analysis: Pearson correlation test.

**Results:** The correlation achieved between the methodologies was 0.97. The good correlation presented highlights the safety of the weighing activity, making the seminal volume verification process even more precise, in addition to guaranteeing the excellence of the institution’s semen analysis.

**Conclusion:** Based on our results, we can confidently affirm that the validation of the seminal ejaculate weighing method using an analytical balance was effective and reliable. These results fully validate implementation in routine practice following WHO recommendations.

**P-22. Successful transfer of mosaic embryo with trisomy of chromosome 14: case report and favorable neonatal outcome**

**J.Boncristiano^1^, E.C Garavini^1^, B.R.L. Moura^1^, J.P.B.M França^1^, E.V Leahy^1^**

*^1^Gerar InVitro*

**Objective:** The aim of this report is to document the treatment, transfer, and birth of an embryo analyzed by Preimplantation Genetic Testing for Aneuploidies (PGT-A). The embryo exhibited low-grade mosaicism, including trisomy of chromosome 14.

**Methods:** A 38-year-old patient sought assistance in June 2022, with a history of endometriosis and low ovarian reserve, initiating treatment. In July of the same year, she underwent the first retrieval, freezing 3 eggs. The following month, another retrieval yielded 6 eggs, all fertilized with her husband’s semen. From this fertilization, 4 blastocysts were obtained for Preimplantation Genetic Testing for Aneuploidies (PGT-A). Results revealed none were euploid, three were aneuploid, and one exhibited low-grade mosaicism, notably trisomy of chromosome 14. Consultations with geneticists were conducted to assess the implications of the transfer. However, the final decision rested with the patient. It is noteworthy that trisomy of chromosome 14 is incompatible with life (Shinawi *et al*., 2008). In born mosaic embryos, common clinical manifestations include dysmorphic craniofacial features, congenital heart defects, and genitourinary abnormalities. Other reported characteristics encompass hypertelorism, body asymmetry, and abnormal skin pigmentation (Shinawi *et al*., 2008; Fagerberg *et al*., 2012; Lynch *et al*., 2004; Von Sneidern & Lacassie, 2008).

**Results:** The patient’s decision to transfer the mosaic embryo resulted in a positive ßhCG test, reaching 2261 mIU/mL. Subsequent ultrasound revealed the presence of a gestational sac with fetal heartbeat. The outcome was the successful birth of a healthy baby on October 12, 2023, weighing 3.960 kg and measuring 51 cm in height. Remarkably, the newborn did not exhibit any of the previously mentioned clinical manifestations, indicating a favorable outcome following the transfer of the mosaic embryo. These results underscore the complexity and variability in the clinical consequences of embryos with low-grade mosaicism, emphasizing the importance of individual considerations and appropriate monitoring when making decisions about embryo transfers.

**Conclusion:** The successful transfer of the mosaic embryo led to the birth of a healthy baby, highlighting the feasibility of individualized choices in such scenarios. This case underscores the importance of careful evaluation, taking into account both genetic results and clinical characteristics, providing valuable insights for guiding future therapeutic decisions in similar cases.

**References**

Fagerberg CR, Eriksen FB, Thormann J, Østergaard JR. Trisomy 14 mosaicism: clinical and cytogenetic findings in an adult. Clin Dysmorphol. 2012;21:45-7.

Lynch MF, Fernandes CJ, Shaffer LG, Potocki L. Trisomy 14 mosaicism: a case report and review of the literature. J Perinatol. 2004;24:121-3.

Shinawi M, Shao L, Jeng LJ, Shaw CA, Patel A, Bacino C, Sutton VR, Belmont J, Cheung SW. Low-level mosaicism of trisomy 14: phenotypic and molecular characterization. Am J Med Genet A. 2008;146A:1395-405.

von Sneidern E, Lacassie Y. Is trisomy 14 mosaic a clinically recognizable syndrome?--case report and review. Am J Med Genet A. 2008;146A:1609-13.

